# Comparison of two electronic hand hygiene systems using real-time feedback via wireless technology to improve hand hygiene compliance in an intensive care unit

**DOI:** 10.1017/ash.2022.270

**Published:** 2022-07-25

**Authors:** José R. Generoso, Eduardo Casaroto, Ary Serpa Neto, Marcelo Prado, Guilherme M. Gagliardi, Fernando Gatti de Menezes, Priscila Gonçalves, Fábio Barlem Hohmann, Guilherme Benfatti Olivato, Gustavo Potratz Gonçalves, Andréa Marques Pereira, Nathalia Xavier, Marcelo Fernandes Miguel, Elivane da Silva Victor, Michael B. Edmond, Alexandre R. Marra

**Affiliations:** 1 Instituto Israelita de Ensino e Pesquisa Albert Einstein, Hospital Israelita Albert Einstein, São Paulo, São Paulo, Brazil; 2 Monash University, ANZIC-RC, Melbourne, New South Wales, Australia; 3 Universidade de São Paulo, São Carlos, São Paulo, Brazil; 4 Infection Control Unit, Hospital Israelita Albert Einstein, São Paulo, São Paulo, Brazil; 5 West Virginia University School of Medicine, Morgantown, West Virginia, United States; 6 Department of Internal Medicine, University of Iowa Carver College of Medicine, Iowa City, Iowa, United States

## Abstract

**Background:**

Most hand hygiene (HH) intervention studies use a quasi-experimental design, are primarily uncontrolled before-and-after studies, or are controlled before-and-after studies with a nonequivalent control group. Well-funded studies with improved designs and HH interventions are needed.

**Objectives:**

To evaluate healthcare worker (HCW) HH compliance with alcohol-based hand rub (ABHR) through direct observation (human observer), 2 electronic technologies, a radio frequency identification (RFID) badge system, and an invasive device sensor.

**Methods:**

In our controlled experimental study, 2,269 observations were made over a 6-month period from July 1 to December 30, 2020, in a 4-bed intensive care unit. We compared HH compliance between a basic feedback loop system with RFID badges and an enhanced feedback loop system that utilized sensors on invasive devices.

**Results:**

Real-time feedback by wireless technology connected to a patient’s invasive device (enhanced feedback loop) resulted in a significant increase in HH compliance (69.5% in the enhanced group vs 59.1% in the basic group; *P* = .0001).

**Conclusion:**

An enhanced feedback loop system connected to invasive devices, providing real-time alerts to HCWs, is effective in improving HH compliance.

Hand hygiene (HH) is considered a category 1A (strongly recommended) intervention; however, solid evidence for this designation is relatively weak (category 2) and is derived from historical cohort studies.^1^ An evidence designation of category 2 highlights the need for more rigorous methodologies when testing HH efficacy. Nevertheless, because of cost and other logistical barriers,^2^ most HH intervention studies use a quasi-experimental design, are primarily uncontrolled before-and-after studies,^3^ or are controlled before-and-after studies with a nonequivalent control group.^4^ These designs do not have a standard nomenclature and are hampered by confounding, selection biases, and regression to the mean.^5^

Effective, locally adapted implementation strategies are increasingly recognized as critical for the maintenance of high levels of HH compliance.^6^ Although direct observation has been considered the gold standard, only a small fraction of HH opportunities can be observed.^7^ Studies employing direct observation are likely to be biased by the Hawthorne effect, which causes behavioral changes among participants of epidemiological studies or infection control interventions.^8^

Automated HH counting through a radio frequency identification (RFID) operating device is an important tool for collecting data about HH, giving us the possibility to provide real-time feedback to healthcare workers (HCWs) about their HH performance.^7–9^ Electronic HH systems are designed to ensure that HCWs perform HH prior to patient care, and some have the capability of issuing an automated alert for HCWs to do so.^10^ However, these systems have not resulted in significant improvement in HH,^11^ even though feedback loops are generally profoundly effective tools for changing human behavior.^12,13^ Other recent studies using electronic HH systems reported significant improvement in HH performance rates or high performance rates.^14–19^ In this study, we evaluated HCW compliance with HH using an alcohol-based hand rub (ABHR) through direct observation (human observer) and with the addition of 2 electronic feedback interventions.

## Methods

This study was conducted in the intensive care unit (ICU) of a private, tertiary-care hospital, Hospital Israelita Albert Einstein, in São Paulo, Brazil, over a 6-month period (July 1–December 31, 2020). The study was approved by the hospital’s institutional review board. This ICU is a 40-bed, medical-surgical unit with all private rooms. We selected 4 rooms (rooms 1–4) in unit 18 for the study interventions. Each room had dedicated supplies for patient care, such as stethoscopes and thermometers. This area has 6 sinks (1 per room and 2 outside the rooms), and the staff consists of 1 nurse, 1 physician, 1 respiratory therapist, and 4 nurse assistants. All the staff has patient contact over their shift and daily duties. Researchers did not previously selected ICU staff who would participate in the study and had no influence over their work schedule.

**Fig. 1.**
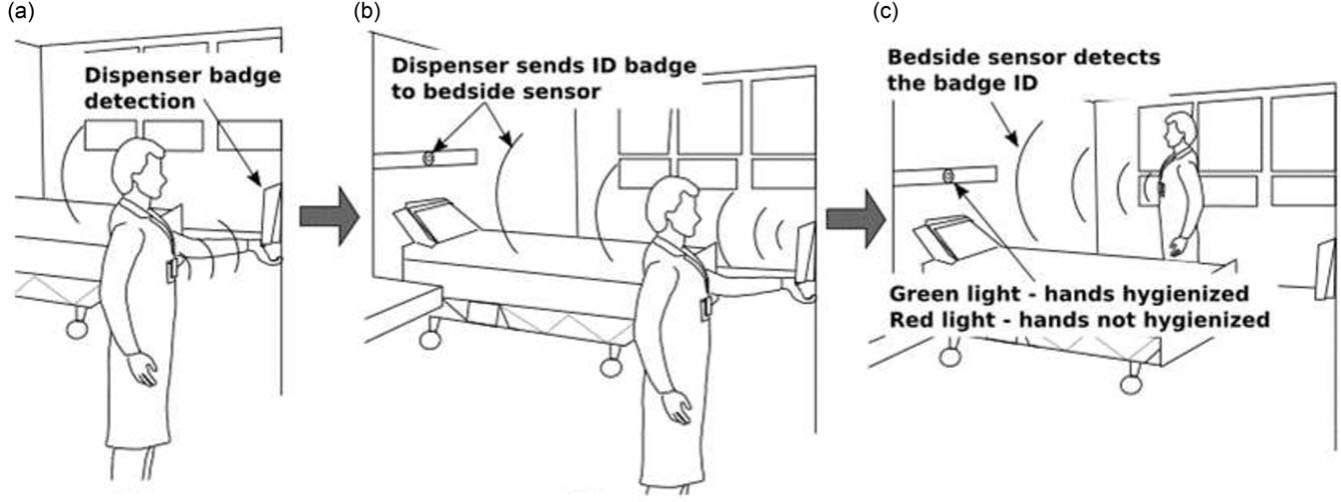
Real-time feedback loop system for hand hygiene monitoring. *Adapted from Marra and Edmond.^20^

The basic feedback loop system was installed in all 4 rooms (Figure 1), and HCWs wore radio frequency identification badges. If HH was performed, HCWs were alerted by a green flashing light above the head of the bed. If HH was not performed, they received an alert with a red flashing light. Rooms 3 and 4 also had invasive-device sensors installed that monitored physical contact by the HCW with the central venous line or urinary catheter (enhanced feedback loop). Similar to the feedback loop that alerts HCWs about HH, the invasive-device sensors flash a green light when an HCW handles the intravenous or urinary catheter to remind them to perform HH. Any time that drugs were administered (ie, the intravenous line, urinary catheter, or urinary bag were manipulated), the sensor would capture its movement and provide the HCW a luminous alert to perform HH.

**Fig. 2.**
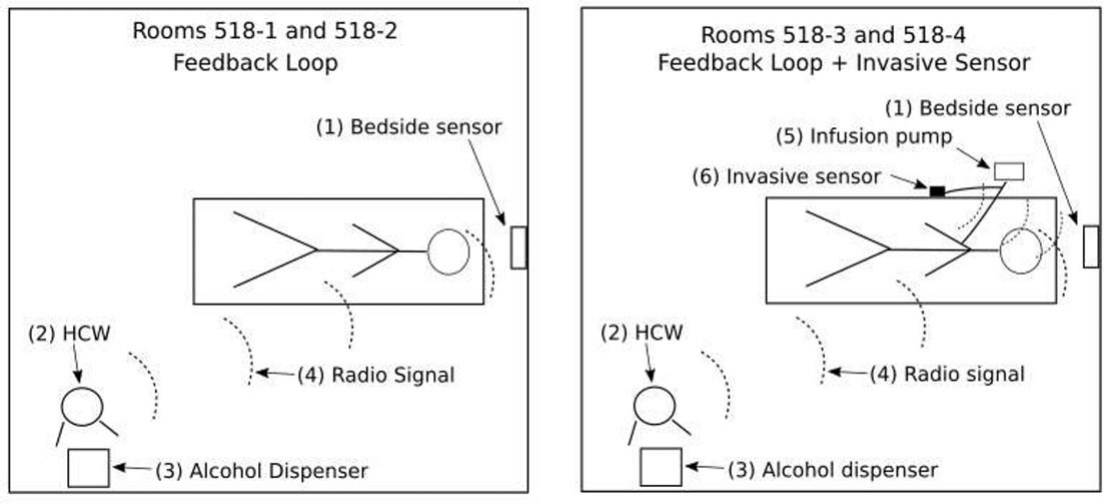
Differences between the devices installed in the ICU rooms. Rooms 3 and 4 have the invasive device sensor, which was not present in rooms 1 and 2 where only the feedback loop system was installed.

The measurement of HH compliance was compared by direct observation, electronic capture of HH events by a system (Infectrack, iHealthSys, São Carlos, São Paulo, Brazil) that utilizes RFID badges and a system connected to an invasive-device sensor. Differences between these electronic technologies are illustrated in Figure 2 HH observations performed by both human observers were performed in all 4 rooms.

For direct observation, the concordance of HH observations between the 2 ICU physicians and the infection preventionist (IP) was established in ICU rooms that were not part of our study by having ICU physicians and the IP observe HH performance in the same unit at the same time and comparing their measured rates of HH compliance.^13^ The ICU physicians (not on clinical duty) were directed to perform 15-minute HH observations in the 4 ICU study rooms (only 1 room per day) at different times between 8:00 a.m. and 8:00 p.m. (morning, afternoon, and night) randomly on weekdays, weekends, and holidays. The 2 ICU physicians recorded the observed HH events (the dispenser activations) and HH opportunities on an iPhone using the iScrub app. After collection, data were organized in an Excel file (Microsoft, Redmond, WA) and sent by e-mail via Wi-Fi^21^ to the project engineer. The 2 ICU physicians counted only HH opportunities within the care process when HH must be performed, as specified by the indications according to the World Health Organization My Five Moments for HH.^22^

### Electronic devices (Infectrack)

Hand hygiene events were registered by electronic HH counter devices with Purell Hand Instant Sanitizer (70% ethyl alcohol + 4% isopropyl alcohol 1-L bag; Gojo, Akron OH). The ABHR dispenser (NXT 1-L model) registers only 1 event every 2 seconds, even if >1 aliquot is dispensed. The dispensers provide the same volume of product per use (∼1.3 mL) and are located inside patient rooms. All dispensers were of the same type (1 L). The nozzles of the dispensers did not clog because every 48 hours a nurse (not on clinical duty) checked them or changed the empty ones in all 4 studied ICU rooms. This nurse also checked the numbers inside the counters. There were 2 ABHR dispensers in each room and 1 between each room.

### Electronic feedback technology

The basic technology uses a wireless identification device (badge) for recording when an HCW performs HH with ABHR using an electronic dispenser inside the patient’s room. Identification devices use ZigBee technology (iHealthSys, São Carlos, São Paulo, Brazil, wireless communication protocol based on the Institute of Electrical and Electronics Engineers standard 802.15.3).^23^

Badges were color coded for HCW category (ie, nurse, respiratory therapist, physician, or nurse assistant), which allowed us to determine which HCW was being observed and performing patient care for any given HH opportunity. A bedside sensor flashed a red light above the patient’s bed when an HCW approached it if HH was not performed. The same bedside sensor flashed a green light if HH was performed. Thus, HCWs were provided with real-time feedback on HH compliance. Database integration allowed reports to be generated demonstrating how many HCWs entered the rooms and performed HH, as well as how many patients were provided care by individual HCWs. Data for HH events were recorded by electronic ABHR counters in alcohol dispensers. The system was activated at the same time the HCW pressed the ABHR dispenser for HH. If multiple HCWs were inside the patient’s room, the system captured 1 HH event at a time by any of those present with an RFID badge. The software allowed analysis of the radio signal strength over a certain time interval, and badge detection was properly achieved without interference in the adjacent bed. The system monitored the real-time signal strength of the radio signal and, by statistical analysis, determined whether the person wearing a badge was within the limits of the surrounding patient area. The range of the bedside sensor was limited to 3 m to avoid an interfering signal from the adjacent bed (in which an HCW wearing a badge could be at a distance <3 m), and a physical barrier was put behind the radio face plate of the bedside sensor.

Figure 1 shows the sequence of steps for detecting HH performance by HCWs. These sensors monitored when HCWs had physical contact with the invasive device (Figure 2). Similar to the feedback loop system that alerted HCWs about HH, the invasive-device sensors flashed a green light when HCWs handled the invasive device, with the intent to remind them to embrace a HH opportunity.

The invasive-device sensor technology utilizes a semirigid cable made of a nontoxic plastic material that transmits the movement of the invasive device to the sensor, which has an internal accelerometer and a microcontroller board for accelerometer data acquisition. These data are then transmitted via radio frequency ZigBee protocol (and 2.4 GHz radiofrequency) to the same bedside sensor used in the basic feedback loop system. Subsequently, the bedside sensor sends the data via radio frequency to a computer in the ICU. Software calibrations were performed to filter patient movements while capturing the movements of HCWs manipulating the invasive device.

**Fig. 3.**
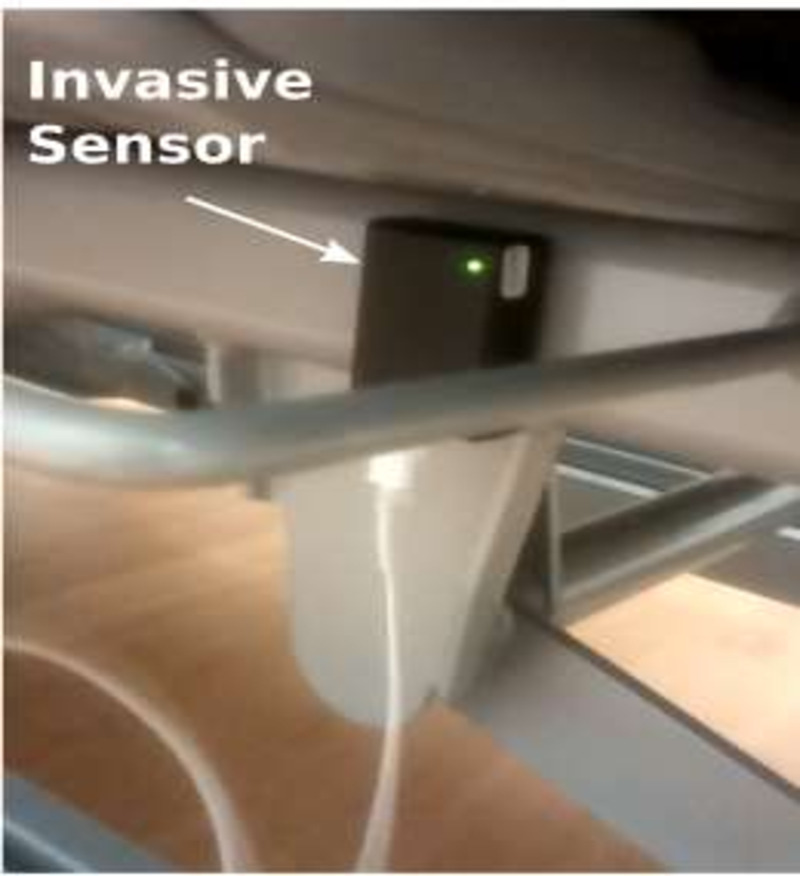
Invasive device sensor flashing the green light when HCW manipulates the invasive device.

**Fig. 4.**
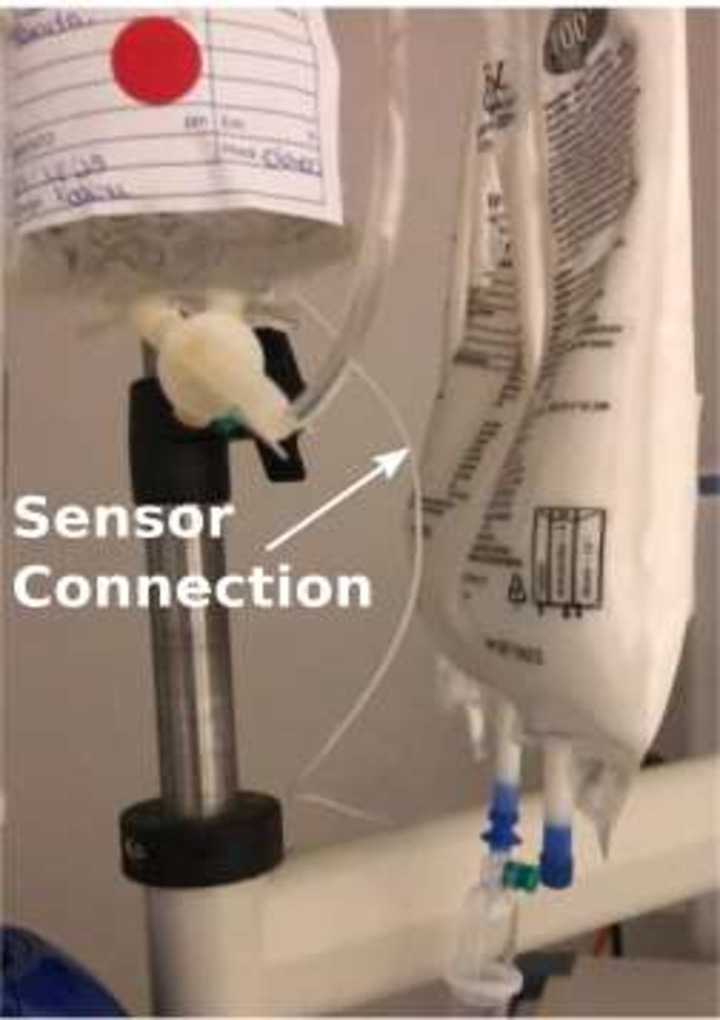
Invasive device sensor connection to the IV bag on a pump.

Figure 3 shows the invasive-device sensor flashing the green light when the HCW manipulated it. Figure 4 shows the semiflexible cable of the invasive-device sensor connected to the IV bag on a pump connected to the central-venous line.

### Statistical analysis

We compared HH compliance between the intervention rooms and the control rooms. Comparisons were made in 2 ways: (1) the number of compliant HH events and the number of HH opportunities recorded by observers, which is required for estimated compliance rates and (2) the number of HH events (HHEs) recorded by the electronic alcohol gel dispenser per patient days. The number of patient days was calculated based on the total number of patients admitted to the 4-room ICU (unit 18) over a 180-day period. The total number of patient days considered for each group was half of the number of patient days of unit 18. We also considered the assessment of agreement between the observers using the κ coefficient with the 95% confidence interval (95% CI).

### Sample size for comparing groups

We estimated 400 opportunities to detect a difference of 15% HH compliance between the intervention and control rooms, assuming a power of 90%.

### Group analysis

Qualitative analyses were described by absolute frequencies and percentages of HH compliance. The quantitative variables were described by medians and quartiles in addition to minimum and maximum values due to the asymmetry observed in the distribution. To evaluate the concordance between measurements, we used dispersion plots and intraclass correlation coefficients. The comparison between different patient rooms for quantitative measurements was made using Mann-Whitney tests. To compare the results from different rooms we used the categorical variables (ie, days of week, shift time of day, and HH opportunities based on My Five Moments for HH) and χ^2^ tests. The analyses were conducted using SPSS version 24.0 software (IBM, Armonk, NY). All tests of statistical significance were 2-sided, with a significance level set at *P* ≤ .05.

## Results

### Comparison of agreement between the HH observers

Analyzing the data recorded by these 2 physicians (HH observers), we obtained a κ value of 0.97 (95% CI, 0.93–1.01; *P* < .001).

### Comparison of rooms in relation to the number of HH per patient day from July to December 2020

We obtained the number of HH events (HHEs) for unit 18 (the unit with rooms 1–4 only) as a whole and the number of patient days also for the groups. We calculated the number of HHEs per patient day using the same denominator divided by 2, both for rooms 1 and 2 and for rooms 3 and 4.

Overall, 50,340 HHEs were recorded in 522 patient days, which resulted in a total of 96.4 HHEs per patient day. In beds 1 and 2, the total number of HHEs was 27,047 with 261 patient days, for an estimated 103.6 HHEs per patient day. In rooms 3 and 4, the total number of HHEs was 23,293 with 261 patient days, for an estimated 89.2 HHEs per patient day.

To compare rooms 1 and 2 versus 3 and 4, we considered the monthly observations per group (6 months) and performed a Mann-Whitney test for independent samples. The median of HHEs per patient day for rooms 1 and 2 was 108.9 (interquartile range [IQR], 66.2–155.4) and for rooms 3 and 4 the median was 91.7 (IQR, 79.3–97.6), with no significant differences between the groups (*P* = .5887).

### Comparison of rooms 3 and 4 (enhanced feedback loop system) with rooms 1 and 2 (basic feedback loop system)

For the total study period, we identified 2,269 HH opportunities with room identification. HH was performed in 59.1% of the opportunities in rooms 1 and 2 (control rooms) and in 69.5% of the opportunities in rooms 3 and 4 (intervention rooms), demonstrating evidence of a significant difference (*P* < .0001) (Table 1). When evaluating subgroups, we also detected differences between groups in the same way (greater adherence in rooms 3 and 4) on weekdays (*P* < .0001) and for the 3 periods of the day: *P =* .0044 in the morning; *P =* .0059 in the afternoon; and *P =* .0015 at night. Regarding “My Five Moments for HH,” we detected significant differences after exposure to body fluid (*P =* .0010) and after touching the patient’s surroundings (*P =* .0068).

**Table 1.** Hand Hygiene Direct Observations in the Control Group (Rooms 1 and 2) and the Interventional Group (Rooms 3 and 4)

Variable	Hand Hygiene Compliance		*P* Value^a^
	Rooms 1 and 2 (N=1,485), No./Total (%)	Rooms 3 and 4 (N=784), No./Total (%)	
Global	877/1,485 (59.1)	545/784 (69.5)	<.0001
**Day of the week**			
Weekday	836/1,431 (58.4)	451/641 (70.4)	<.0001
Weekend (Saturday or Sunday)	41/54 (75.9)	94/143 (65.7)	.1694
**Time of day**			
Morning (07:00–12:59)	406/642 (63.2)	236/326 (72.4)	.0044
Afternoon (13:00–18:59)	301/526 (57.2)	255/385 (66.2)	.0059
Night (19:00–06:59)	170/317 (53.6)	54/73 (74.0)	.0015
**Hand hygiene opportunity (My Five Moments for Hand Hygiene)**			
After body fluid exposure/Risk	25/45 (55.6)	50/59 (84.7)	.0010
After touching a patient	264/336 (78.6)	201/249 (80.7)	.5240
After touching patient surroundings	256/542 (47.2)	125/215 (58.1)	.0068
Before clean/Aseptic procedures	13/31 (41.9)	10/20 (50.0)	.5720
Before touching a patient	319/531 (60.1)	159/241 (66.0)	.1178

a χ^2^ test.

## Discussion

In this study, we have demonstrated that the use of real-time feedback by wireless technology connected to a patient’s invasive devices resulted in a significant increase in HH compliance. Measuring HH adherence is an important but complex activity for which little specific methodological guidance is available.^24^ Human HH observation may not report accurate measures of HH compliance.^14^ Introducing electronic monitoring of HH can led to significant improvements in HH performance for a long period.^17^ It can also result in reductions in hospital associated infections.^17,18^ In addition, automated HH monitoring can be useful to monitor HH compliance at individual and group levels, which increases the understanding of HH compliance behavior.^16,19^ Various measuring methods can be used that all have inherent advantages and disadvantages, as well as underlying assumptions.^3,24^ It is important to realize that behavior may change over time, and each of the methods can result in adherence rates that are valid for the research period only.^24^

As a continuous educational method, displaying posters with gain-framed messages or messages emphasizing the positive outcomes of HH compliance are theoretically effective in motivating HH procedures^25^ and may promote it daily. An interrupted time-series analysis conducted in a neonatal intensive care unit reported a positive outcome of gain-framed messages in the frequency of hand disinfection events and compliance using electronic devices in ABHR dispensers.^26^ Implementing an electronic HH monitoring system includes a collaborative environment using data to drive improvement, consistent and constant messaging, and staff empowerment.^15^

In many studies, a multimodal strategy involving feedback of local data on healthcare-associated infections and HH practices is an essential element for motivating staff to improve their performance^27–29^ and to sustain HH compliance with a shared accountability model.^30^ A widely used strategy involves setting high and maybe unachievable targets (eg, 90%–100% compliance); failures to meet these rates results in scrutiny and sometimes substantial penalties.^26^ Punishing hospitals that do not achieve unrealistic targets is likely to be detrimental to the broad enterprise of infection prevention.^31^ Hospital institutions should make decisions about which approach to use for HH audit because each has limitations. The presence of HH observers is very likely to increase HH frequency and to overestimate HH compliance, but relying solely on product uptake or electronic counting devices results in loss of information because these methods provide no information about the HH event in the context of care delivery.

Our study had several limitations, but we pursued a novel approach to compare different types of feedback to identify which is more effective.^3^ Notably, these technologies have remained limited in use because they are expensive and generate high maintenance costs, even though it is likely that the cost will decrease over time. However, having different technologies for measuring compliance allows hospitals to choose among tools with different advantages and disadvantages and to combine them with standard approaches. Many articles about automated HH monitoring systems report HH performance rates in a similar way, as an HH observation with the HH events per number of HH opportunities ×100 instead of HH events per patient day.^17–19^ However, the number of HH opportunities reported by those studies could not be accurate as denominator (HH opportunities) for HH compliance rate if only the number of room entries (proxy moment 1) is considered because leaving the bed space might be considered similar to room exit (proxy for moments 4 and 5). Many opportunities exist for HH, and when moments 2 and 3 are not considered as a denominator number, using those numbers (entry into and exit from the room) could produce a spurious rate if the number of HHE is greater than the number of HH opportunities. We had concerns about this situation because the ratio of ABHR used to nurse visit can be almost 3 (ie, HHEs per HH opportunities = entry + exit nursing visits), depending on the success of HH intervention.^32^ Thus, we chose to show the automated HH monitoring rates as HH events per patient day. Also, our study was affected by the COVID-19 pandemic outbreak in Brazil when access to ICU was limited and researchers were not allowed in the rooms to collect data. This situation could have affected our low number of moment 2’s (before clean and aseptic procedure) and moment 3’s (after body fluid exposure or risk) of the “My Five Moments for HH.” The green color for the flashing light in our enhanced feedback loop system may have been interpreted by some HCWs as a “go green” sign rather than actually alerting them to comply with HH protocols.

In conclusion, an enhanced feedback loop system connected to invasive devices that provides real-time alerts to HCWs is effective in improving HH compliance. Potential challenges are staff use of badges and remote data collection tools. Further studies evaluating behavior changes regarding HH and hospital-associated infections before and after a feedback loop system installation are needed.
